# Issues in design and interpretation of MDR-TB clinical trials: report of the first Global MDR-TB Clinical Trials Landscape Meeting

**DOI:** 10.1186/1753-6561-9-S8-S1

**Published:** 2015-11-06

**Authors:** Carole D Mitnick, ID Rusen, Lisa J Bain, C Robert Horsburgh

**Affiliations:** 1 Department of Global Health and Social Medicine, Harvard Medical School, Boston, MA, USA; 2 International Union Against Tuberculosis and Lung Disease, New York, NY, USA; 3 Independent Science Writer, Elverson, PA, USA; 4 Department of Epidemiology, Boston University School of Public Health, Boston, MA, USA

## Abstract

Recognizing that the current MDR-TB regimen is suboptimal and based on low-quality evidence, the Global MDR-TB Clinical Trials Landscape Meeting was held in December, 2014 to strategize about coordination of research and development of new treatment regimens for this disease that affects millions of people worldwide every year. Sixty international experts on multidrug-resistant tuberculosis (MDR-TB) met in Washington D.C. and Cape Town, South Africa to consider key MDR-TB trial-related issues, including: standardization of definitions; clinical trial capacity building and; regimens optimized to foster compliance, avoid the emergence of resistance and have clinical relevance for special populations, including children and those co-infected with HIV. Underpinning all of this is the generation of a sufficient evidence base to facilitate regulatory approval and improved normative guidance. Participants discussed treatment combinations currently being studied in Phase 2B and Phase 3 trials as well as other promising new regimens and combinations that may be evaluated in the near future. These include regimens designed specifically to enable shorter duration and all-oral treatment as a means of maximizing treatment completion. It is hoped that clear definition of these challenges will facilitate the process of identifying solutions that accelerate progress towards effective, non-toxic treatments that can be programmatically implemented.

## Introduction

According to the World Health Organization (WHO), an estimated 9 million people developed tuberculosis (TB) in 2013, and 1.5 million died from the disease [1]. Although the rate of TB has declined modestly in recent years as a result of effective diagnosis and treatment, multidrug-resistant TB (MDR-TB) – i.e., TB resistant to the two most effective first-line drugs, rifampicin and isoniazid – has not declined and remains a significant global challenge. The WHO estimates that MDR-TB is responsible for 3.5% of new and 20.5% of previously-treated TB cases. Globally, approximately 25% of TB-related mortality is due to MDR-TB. Additionally, about 9% of MDR-TB patients have extensively drug resistant TB (XDR-TB), meaning their TB is also resistant to at least two other second-line drugs.

Treatment of MDR-TB requires longer regimens – at least 20 months – with lower success rates compared to the treatment of drug sensitive TB (DS-TB) [2]. These longer regimens, along with a need for enhanced laboratory testing and hospitalization, increase the cost and complexity of MDR-TB treatment. The impact of these differences is particularly profound in high-burden countries such as South Africa [3]. Drug resistant TB also threatens the lives of healthcare workers in high-burden countries [4]. The current MDR-TB regimen is suboptimal for many reasons: it is based on poor evidence of efficacy [5], is too long and toxic, includes injectable drugs that add cost and discomfort, and does not reflect evolving drug resistance patterns.

In the past decade, shorter duration regimens for the treatment of DS-TB have been explored, and two new drugs specifically developed for MDR-TB – bedaquiline (BDQ) and delamanid (DLM) – have recently been introduced. There is however, little evidence about their optimal use. There are also other new drugs in development as well as investigational regimens using combinations of new and repurposed drugs [6]. Clinical trials of a number of experimental regimens against MDR-TB are currently underway or being planned (Table 1).

**Table 1:** Summary of Experimental Regimens in MDR-TB Clinical Trials Underway or Planned

Trial Name (funding source)	Duration of Experimental Regimen (months)	Comparator	Experimental Arm(s)	GOAL			
				
				Shorten	All-oral	Improve tolerability	Improve cure rates
C 213/Phase 3 Delamanid (Otsuka)	24	WHO Std	DLM + OBT				X

NeXT (MRC-SA)	6-9	SA Std	BDQ+LZD+LFX+ETA/INH_H_+PZA	X	X		X

endTB (PIH-MSF)	9	WHO Std (+/- new drug)	BDQ+LZD+MXF (HD)+PZA BDQ+CFZ+LZD+LFX+PZA DLM+LZD+MXF (HD)+PZA DLM+CFZ+LZD+LFX+PZA DLM+CFZ+MXF+PZA	X	X	X	X

TB-PRACTECAL (MSF)	6	WHO Std (+/- new drug)	BDQ+PRT+LZD+MXF BDQ+PRT+LZD+CFZ BDQ+PRT+LZD	X	X	X	X

STREAM Stage 1 (USAID+)	9	WHO Std	CFZ+EMB+MXF+PZA+4(KM+INH_H_+PTO)	X			X

STREAM Stage 2 (USAID+)	6: 9:	WHO Std 9 mo. Regimen	BDQ+LFX+CFZ+PZA+2(INH_H_+KM) BDQ+CFZ+EMB+LFX+PZA+4(INH_H_+PTO)	X	X		X

NC-005* (GATB)	2	None for MDR Arm	BDQ+PRT+MXF+PZA *Note: this is a phase 2 study in DS & MDR-TB*	X	X	X	X

NiX-TB	6-9	None	BDQ+PRT+LZD	X	X	X	X

STAND* (GATB)	4-6	None for MDR Arm	PRT+MXF+PZA	X	X	X	X

Novartis	24	WHO Std	CFZ+OBT				X

Opti-Q	6	LFX 11 mg/kg + OBT	LFX 14, 17 ands 20 mg/kg (all + OBT)				X

BDQ - Bedaquiline

CFZ - Clofazimine

DLM – Delamanid

EMB – Ethambutol

INH - Isoniazid

LFX - Levofloxacin

LZD – Linezolid

MXF – Moxifloxacin

PRT- Pretomanid (PA-824)

PZA - Pyrazinamide

OBT= optimized background therapy

HD=high dose

*Primarily DS-TB study with nonrandomized comparator MDR-TB arm; patients with PZA or MXF resistance excluded

Recognizing the unique opportunity presented by these recent, exciting developments, and the synergies that could be realized through consensus and coordination among stakeholders involved in these MDR-TB trials, TREAT TB (Technology, Research, Education, and Technical Assistance for Tuberculosis) – a USAID-funded, Union-implemented research initiative – and RESIST-TB (Research Excellence to Stop TB Resistance) co-hosted the first Global MDR-TB Clinical Trials Landscape Meeting in December, 2014. The meeting was held simultaneously in Washington, D.C. and Cape Town, South Africa. Key issues addressed included composition of regimens, methodological challenges for evaluating new drug regimens, standardization of trial definitions, and mechanisms for communication and coordination among trials groups.

## Overarching issues in MDR-TB trial design

Clinical trials to evaluate drugs and regimens for treatment of MDR-TB are more complex and less easy to standardize than clinical trials of drug-susceptible TB. These complexities result in some issues specific to designing MDR-TB trials include:

• Weight of evidence required to change policy and practice. For example, given the urgent need to develop MDR-TB regimens, it might be justifiable to accept one small, well-controlled RCT, or make concessions with regard to the choice of control, statistical adjustment for multiplicity, etc.

• Maintaining flexibility within the trial design so that it can be adapted to external data developed while the trial is in progress, thus ensuring that trial results retain relevance when they are published. Bayesian adaptive randomization is one possible approach to the design of efficient multi-arm trials [7]. This approach has been used most often in oncology trials. Initial randomization probabilities are fixed among arms. Probability of a treatment effect estimated through interim analysis of data (sometimes using a surrogate marker) affects subsequent assignments. This enables 1) increased randomization to study arms that show improved performance, and 2) discontinuation of arms that are ineffective or unsafe. Compared to fixed randomization, Bayesian adaptive randomization results in a reduction in overall sample size requirements to detect a specified effect size.

• Selecting the appropriate patient population. For MDR-TB regimens to be effective on a population level, they will need to work across a range of patterns of resistance to anti-TB drugs and of co-morbidities. Study populations will need to reflect this heterogeneity so eligibility criteria should be minimally restrictive.

• Choice of control regimen. Although the internal control is considered fundamental to the randomized trial, there is currently no universal standard of care, and treatment standards may change over time. Control options might include the locally used regimen based on the WHO guidelines or (a variant of) one of the shortened regimens that has yielded success in Bangladesh and West Africa [8-10].

• Duration and frequency of follow up. Introduction of new regimens entails concerns about possible toxicity and, for shortened regimens, increased risk of relapse. Establishing standard or minimum duration and frequency of follow up is necessary to produce comparable results across trials. Since regimens being evaluated are of different durations, selection of a fixed amount of time for post-treatment follow up may not permit comparison of regimens within and between trials. Establishing a minimum duration of follow up relative to time since randomization—rather than after treatment—may represent the most practical solution. There are, however, limited data on the timing of relapse after treatment for MDR-TB so the length of this period cannot be determined based on substantial evidence. This issue is discussed in more detail below.

• Safety monitoring. For new drugs, intensive safety monitoring is needed but the interpretation of results of such monitoring must be made in light of the distinctly different risk-benefit balance between efficacy and toxicity compared to existing drugs for DS-TB.

## Regimen design issues

### Shortening the overall duration of treatment

Shortening the duration of TB treatment could dramatically improve adherence and thus minimize the development of resistance, as well as lower the costs of therapy. However, determining the appropriate duration of therapy has proven challenging since the nature of the relationship between bacteriological endpoints in the first 2-3 months of treatment (usually culture conversion on solid media) and long-term outcomes is poorly understood. Murine relapse studies and stratified approaches also have thus far failed to clarify treatment duration for MDR-TB. In general, MDR-TB regimens are less potent than DS-TB regimens and require more time to achieve stable culture conversion. Most data on treatment response in MDR-TB have been obtained in observational cohort studies. A recent meta-analysis of these studies found that treatment duration of 7-8.5 months in the initial phase, with a total duration of 25-27 months yielded the highest odds of success [11], yet the authors of this study emphasized the “serious limitations” of these observational data and the urgent need for RCTs that assess the optimum treatment duration. Establishing a common global framework that encompasses data from DS and MDR-TB clinical trials in order to identify intermediate endpoints would enable comparison of drug class-specific effects and the efficacy of different regimens. Although two- or three-month culture conversion has shown promise as a possible surrogate endpoint, variability among different studies suggests that a summary measure of culture conversion over time may hold more potential as a surrogate [12].

While shorter duration treatment may be sufficient for the majority of patients, a minority of patients will relapse. However, it may be possible to identify subpopulations likely to relapse by mining existing datasets and analyzing new studies to determine baseline and on-treatment characteristics, and interim endpoints that correlate with relapse. These data may also enable predictive modeling. Unfortunately, currently available retrospective observational data sets disproportionately contain data on patients treated for 20-24 months, and thus provide little data on whether shortened regimens might have acceptable relapse rates.

Conversely, children, and other sub-populations prone to have lower bacillary burdens, could be particularly well suited for shortened regimens. Since current regimens—including presentation and dosing—are not optimized for children, it would be particularly valuable to concurrently explore treatment shortening for pediatric patients and for adults.

It may also be possible to optimize treatment regimen concurrently with shortening the duration of treatment. Innovative trial designs, e.g., adaptive designs, may facilitate such studies, but will require additional capacity. Any studies should also be accompanied by cost analyses, as well as other policy-related efforts to ensure uptake of shorter regimens.

### Eliminating injectable agents from the regimen

Current recommended MDR-TB treatment regimens require daily painful injections for 8 months, requiring considerable infrastructure and investment in nurse and patient time; associated ototoxicity is substantial and irreversible. Moreover, resistance to injectable drugs (kanamycin, amikacin, and capreomycin) is increasing [13]. All-oral treatment offers potential advantages in terms of decentralized, community-based delivery and fewer injection-related adverse events [6]. The opportunity to provide all-oral treatment is now possible with the recent introduction of DLM and BDQ. Current constraints to their use include the fact that these drugs have yet to be optimized within a background regimen. Safety concerns about the combination of BDQ and DLM will be addressed with a combination PK study since both classes of drugs have been associated with QT prolongation. Nevertheless, other advances with existing oral drugs—such as late-generation fluoroquinolones, clofazimine (CFZ), linezolid (LZD), and pyrazinamide (PZA)—including increased dosing and shortened regimens, also support the potential for effective all-oral regimens.

The endTB project (Expand New Drug Markets for TB) is planning a phase III randomized (adaptive) open-label trial to optimize all-oral regimens by combining BDQ or DLM with CFZ, LZD, a fluoroquinolone (moxifloxacin or levofloxacin) and PZA. endTB will test regimens with one new drug; further investigation of combinations with both BDQ and DLM may be indicated after the aforementioned combination study (NIAID, ACTG Protocol A5343). In endTB, subjects will be randomized to 6 arms. Interim analyses will guide adaptation of randomization, favoring most effective arms. Study assessments will strongly emphasize safety. One important issue that arose in study design was the choice of control (see “Next steps to advance regimen development for MDR-TB”).

Questions persist about the best oral agents to replace injectables since the quality of evidence is low supporting the role of injectables in MDR-TB treatment. Moreover, there is heterogeneity in the response to these drugs. Data from MDR-TB observational cohorts may also be mined to provide guidance for optimal design of injectable-free regimens.

New animal studies could inform the substitution choices, by further elucidating the mechanisms of action of injectable and oral agents. Eliminating injectable agents, it was noted, may not be feasible for all patients. Options could be considered such as the use of alternative routes of delivery of these drugs (e.g., inhalation) or shortening the period of injectable administration when a new or repurposed drug is added to the regimens.

## Provision of adequate evidence

WHO’s mandate to provide normative guidance for countries has been reflected in a number of recent documents. In 2012, a strategic roadmap was developed by WHO to guide the global development of new drugs and drug regimens for the treatment of DS and DR-TB. WHO subsequently developed a policy implementation package (PIP) to guide introduction of new TB drugs and two interim guidance documents on the use of BDQ (June 2013) [14] and DLM (Oct 2014) [15] in the treatment of MDR-TB. These documents address key issues regarding the use of new drugs and regimens: what is the added value and anticipated impact of the new drugs or regimens, what populations would most benefit, and what are the possibilities for optimal deployment of these new drugs or regimens in various countries. Designs of trials to evaluate new drugs and regimens must ensure that the evidence they generate will provide an adequate basis for approval by regulatory agencies and practice guideline panels.

In recognition that a series of trials have been initiated or are about to start investigating combination regimens, including new or re-purposed drugs, for the treatment of MDR-TB, WHO is preparing an information note on MDR-TB regimen development describing the evidence and trial documentation they will require to develop or update policy recommendations on the treatment of MDR-TB. Data from preclinical, pharmacokinetic/ pharmacodynamic (PK/PD), drug-drug interaction (DDI), and clinical studies will be considered to contribute to the evidence base. Results from these studies will be evaluated using the GRADE (Grading of Recommendations, Assessment, Development, and Evaluation) system [16]. This approach requires evaluation of the study design, number of studies done, and other characteristics in order to determine the quality of evidence and the strength of the recommendation. The information note is currently in draft form, with WHO seeking feedback from experts in the field and drug developers regarding the proposed framework.

## Consideration of specific antimycobacterial agents with likely important roles in new regimens

Prior to the workshop, six anti-tuberculosis drugs were identified as particularly important for improved treatment of MDR-TB. Criteria for selection included evidence of antimycobacterial efficacy, being new (approved or in late stages of clinical development); being drugs to which populations have had limited prior exposure; or being so important to efficacy of current MDR-TB regimens that their exclusion seems ill-advised. The drugs selected for review at the workshop were bedaquiline, fluoroquinolones, delamanid, oxazolidinones, clofazamine, and pretomanid). The advantages and challenges of using these drugs are summarized below.

***Bedaquiline*** (BDQ), an ATP synthase inhibitor, was conditionally approved by the U.S. Food and Drug Administration (FDA) in 2013 and by the European Medicines Agency (EMA) in 2014 for the treatment of MDR-TB. It is active against both DS and MDR strains of *M. tuberculosis*, with strong bactericidal and sterilizing properties. The WHO and CDC recommend its use in patients with MDR-TB who cannot be treated with a minimum of three effective drugs [17]. Common adverse effects include nausea, joint pain and headache. There are also concerns about prolongation of the QT interval on electrocardiogram and hepatic toxicity [18].

A number of unanswered questions about BDQ need to be better understood as the drug becomes more widely used in clinical practice. In particular, non-target-based resistance and efflux-based resistance, as well as cross-resistance with clofazimine (CFZ) may limit the usefulness of BDQ in clinical practice. Resistance is already developing to BDQ, pointing to the need for robust regimens that protect against emergence of resistance and strategies to promote compliance.[19]

***Fluoroquinolones***. Fluoroquinolones (FQs) are a class of broad-spectrum antibiotics that inhibit the DNA gyrase enzyme, which is essential for bacterial replication. They are among the most effective drugs used to treat MDR-TB. While these drugs have excellent early bactericidal activity, they lack sterilizing activity and thus need to be given in combination with sterilizing agents. The class member thought to be most potent (moxifloxacin) can prolong the QT interval, as can other TB drugs of interest for novel regimens. Consequently, their safety in combination therapy must be further evaluated. Resistance and cross-resistance have also emerged as major concerns. In addition, the optimal dose of the most effective fluoroquinolones – moxifloxacin and levofloxacin – has not yet been determined either as single agents, in combination regimens, or for treating latent infections.

Three types of studies were proposed to evaluate the optimal dose of fluoroquinolones in combination with optimal background therapy or with other backbones for the treatment of MDR-TB. An optimal dose study could test fixed doses (e.g., 400, 600, or 800 mg) or mg/kg doses for PK/PD, safety, tolerability, and effectiveness. A combination study would test whether fluoroquinolones can or should be given in combination with other drugs that prolong the QT interval through dose optimization studies with different backbone regimens. The third suggested type of study would evaluate the optimal regimen for prevention of MDR-TB in close contacts of patients with MDR-TB.

***Delamanid*.** Delamanid (DLM) is a novel anti-TB agent of the nitroimidazole class that inhibits the synthesis of mycolic acid, a component of the cell wall and envelope of *M. tb.* It was registered by the EMA, Japan’s PMDA, Korea’s MFDS, and will be submitted to the U.S. FDA in 2015 or 2016 for the treatment of MDR-TB. Two clinical studies of efficacy and safety indicated improved outcomes, including lower mortality, with long-term (≥ 6 months) compared to short-term (≤ 2 months) DLM treatment, when added to optimized background therapy for 12-18 months [20-22]. Recipients of delamanid had more QT prolongation than those receiving placebo. There is no reported cross-resistance to existing anti-TB drugs.

Studies underway will test the effectiveness of DLM combination with moxifloxacin and other agents in HIV uninfected and coinfected patients. A pediatric formulation of DLM has also been developed and is being tested. As noted, ACTG study A5343 will assess drug-drug interactions between DLM and BDQ.

***Oxazolidinones***. Oxazolidinones are protein-synthesis inhibitors that prevent bacteria from growing and reproducing. The first-in-class linezolid (LZD) was approved in 2000 for the treatment of drug resistant bacterial infections and has demonstrated effectiveness in DR-TB. Several new oxazolidinones are in development (sutezolid, AZD 5847, tedizolid, and radezolid.) LZD can be combined with other TB drugs, has high oral bioavailability, pharmacodynamics that permit once-daily dosing, and has a low propensity for resistance emergence [23]. However, long-term use has been limited by toxicity, particularly myelosuppression and myelopathy.

Clinical and non-clinical studies suggest that intermittent dosing could avert LZD-associated toxicity while retaining efficacy. Further investigation is necessary to confirm this relationship and optimize the dosing regimen. Studies are also needed to determine whether higher doses required to suppress resistance could be achieved through intermittent dosing. With regard to new-generation oxazolidinones, murine studies suggest that sutezolid may have more potency against intracellular *M. tb.* and that other drugs in this class have additive effects when combined with regimens including BDQ, PA-824, and PZA [24,25].

***Clofazimine***. Clofazimine (CFZ) is one of the older TB drugs, approved for the treatment of leprosy and more recently, used off-label for MDR-TB [26]. It binds to DNA to disrupt bacterial grown and also has anti-inflammatory activities. It has been tested in several MDR-TB regimens designed to shorten treatment [8-10,27], and is also being included in other treatment shortening programs including the STREAM, TB PRACTECAL and endTB trials (Table 1). Novartis is now designing a study to demonstrate the intrinsic activity of CFZ against *M.tb*., as well as to fill in the gaps in toxicology, safety, PK/PD, mechanisms of action, and drug resistance. There may also be opportunities to use CFZ in pediatric regimens.

***Pretomanid.***Pretomanid (PA-824), a nitroimidazole, is a novel anti-bacterial compound developed by the TB Alliance (TBA) with significant bactericidal and sterilizing activity alone and in combination with BDQ and PZA [24]. It is a pro-drug with a novel, complex, and incompletely understood mechanism of action [28]. Designed to enable a simple dosing schedule, short treatment duration, good side-effect profile, and minimal interaction with anti-retrovirals, it is being tested in multiple MDR-TB regimens including the STAND Phase 3, NC005, and Nix-TB (in patients with XDR-TB) studies (Table 1). PA-824 is also being studied in an MDR-TB treatment trial by Médecins Sans Frontières (MSF), the TB-PRACTECAL study, also shown in Table 1.

Table 1 summarizes experimental regimens for MDR-TB in current or planned clinical trials. These trials have been designed to achieve different goals, e.g., shortening treatment, providing an all-oral regimen, improving tolerability, or improving cure rates.

## Next steps to advance regimen development for MDR-TB

Progress in developing more effective regimens for MDR-TB will require continued international collaboration among stakeholders, an outcome that will hopefully be advanced by the Global MDR-TB Clinical Trials Landscape Meeting. Participants agreed on the need to focus efforts in a number of key areas:

## Standardization of MDR-TB definitions

For harmonization across trials, standardization of interim and post-treatment endpoints is critical. Definitions have been proposed by the WHO [20], FDA [29] and EMA[30], and consensus research outcomes definitions for MDR-TB have been proposed for drug-resistant TB in children [31]. However, there remains a need for universally accepted definitions. A multi-national group of TB clinical trials investigators has proposed consensus definitions for efficacy endpoints (culture conversion, death, unfavorable outcome, favorable outcome, cure, treatment failure, recurrence/reinfection, and recurrence/relapse); tolerance and adherence endpoints (“lost to follow up”, treatment discontinuation/modification, and inadequate adherence); safety endpoints; standard of care; optimized background therapy. The team has also discussed preferences regarding solid or liquid culture, molecular tests, length of follow-up period, managing drugs with long half-lives, predictor variables, and pediatric considerations. A RESIST-TB collaborative group developed a consensus document addressing these issues and this manuscript is currently under review.

## Building MDR-TB trial capacity

In order to be ready for complex combination trials in the future, increased clinical trial capacity—both sites and potential participants—is necessary. Including adolescents in adult trials could be one means of increasing the number of available subjects. Adolescents have adult-type disease and metabolize drugs similarly to adults. Building MDR-TB observational cohorts in a broad range of locations, and establishing networks of sites, could provide trial-ready sites and subjects when needed. Community engagement will be essential to this effort. Efforts should also be made to incentivize treatment programs seeing patients with MDR-TB to participate in prospective MDR-TB observational cohort studies. RESIST-TB has developed site development tools and has volunteered to serve as a repository for observational cohort data.

## Implementing adjunct measures to enhance clinical benefits of introducing new drugs

As new drugs are introduced, it will be important to optimize their use and avoid emergence of resistance through a combination of:

• Development of more robust companion regimens

• Incorporation of efflux pump inhibitors and other novel agents in regimens

• Roll-out of drug susceptibility testing for the new agents

• Trials of preventive therapy for MDR-TB contacts

• Identification of super spreaders [32] with DR-TB, combined with isolation and/or treatment with aggressive regimens for such patients.

• Development and roll-out of rapid tests for drug susceptibility.

• Development of improved outcome measures for comparing the efficacy of regimens in different populations; for example, time to positivity in MGIT (mycobacterial growth indicator tube) and more uniform approaches to the interpretation of chest radiographs.

• Therapeutic drug monitoring for new drugs.

• Development and implementation of strategies to foster adherence to MDR-TB regimens.

## Developing tools to predict failure and relapse

Tools are needed to predict failure and relapse at both the patient level and treatment arm level in clinical trials. For example, change in time to positivity in MGIT combined with baseline bacillary load may identify patients at the highest risk of relapse. Studies have indicated that it is possible to stratify patients into those at very high risk of relapse; however, translating this into an operational strategy remains a challenge. Moreover, predictors of relapse in MDR-TB may not be the same as those in DS-TB.

## Improving outcomes by understanding adherence and social determinants of success

Few, if any, studies build social determinants into clinical trial protocols. This represents a lost opportunity since social factors may explain some of the heterogeneity in treatment outcomes both within and between treatment arms. Gathering such information in the context of trials could provide valuable information about interventions that might optimize treatment outcomes across patients of different socio-economic status.

## Challenges in common control regimens

Several issues emerged with regard to the control regimen. First, since the current WHO regimen is widely perceived as suboptimal, there are ethical concerns about including this as a control. Second, in some countries such as South Africa, BDQ use may soon be increasing for MDR-TB treatment, at least for HIV-negative patients; however, BDQ is still not available in most high-burden MDR-TB countries. Consistency in the selection of controls across sites and trials would enable data to be combined and compared. Given the differences in standard of care in different countries and regions, however, this may not be feasible. Further, the definition of an optimized background regimen may change during the course of a trial, in the face of emerging evidence. The discussions highlighted the obstacles that exist now and likely in the future in identifying a uniform control regimen across trials.

## Treating MDR-TB in special populations, including children and patients co-infected with HIV

Worldwide, about 13% of people with TB are also co-infected with HIV; about 78% of these cases are from the African region [1]. The co-occurrence of these two infections results in more severe disease and increased mortality, yet little is known about the treatment of MDR-TB in the context of HIV infection, including among pediatric patients. Thus, workshop participants agreed on the need to focus research on the implications of co-infection. Identifying and assessing issues specific to pediatric patients is especially needed.

## Conclusions

Meeting participants agreed that the issues discussed at the meeting and summarized in this report need to be pursued, and that agreement on a number of core issues would facilitate making timely progress towards improved treatment and control of the global MDR-TB epidemic. Participants agreed to work together through RESIST-TB (http://www.resisttb.org) and other fora to move the field forward. A follow-up meeting is planned for Spring 2016 to assess progress toward these goals, facilitate continued communication among trials groups and consider additional initiatives to accelerate this process.

## Author contributions

LB prepared the initial draft of the meeting report. CRH, IDR and CM developed the meeting concept, supported meeting implementation and provided critical input and review of meeting report.

## Competing interests

The authors declare that there are no competing interests
